# Efficacy and safety of trastuzumab, lapatinib, and paclitaxel neoadjuvant treatment with or without prolonged exposure to anti-HER2 therapy, and with or without hormone therapy for HER2-positive primary breast cancer: a randomised, five-arm, multicentre, open-label phase II trial

**DOI:** 10.1007/s12282-018-0839-7

**Published:** 2018-02-14

**Authors:** N. Masuda, M. Toi, N. Yamamoto, H. Iwata, K. Kuroi, H. Bando, S. Ohtani, T. Takano, K. Inoue, Y. Yanagita, H. Kasai, S. Morita, T. Sakurai, S. Ohno

**Affiliations:** 1 Department of Surgery, Breast Oncology, NHO Osaka National Hospital, Osaka, Japan; 2 Department of Surgery (Breast Surgery), Kyoto University Graduate School of Medicine, 54 Kawahara-cho, Shogoin, Sakyo-ku, Kyoto, 606-8507 Japan; 3 Division of Breast Surgery, Chiba Cancer Center, Chiba, Japan; 4 Department of Breast Oncology, Aichi Cancer Center Hospital, Nagoya, Japan; 5 Department of Breast Surgery, Tokyo Metropolitan Cancer and Infectious Diseases Center Komagome Hospital, Tokyo, Japan; 6 Breast and Endocrine Surgery, Faculty of Medicine, University of Tsukuba, Ibaraki, Japan; 7 Department of Breast Surgery, Hiroshima City Hiroshima Citizens Hospital, Hiroshima, Japan; 8 Department of Medical Oncology, Toranomon Hospital, Tokyo, Japan; 9 Division of Breast Oncology, Saitama Cancer Center, Saitama, Japan; 10 Department of Breast Oncology, Gunma Prefectural Cancer Center, Gunma, Japan; 11 Institute for Advancement of Clinical and Translational Science, Kyoto University Hospital, Kyoto, Japan; 12 Department of Biomedical Statistics and Bioinformatics, Kyoto University Graduate School of Medicine, Kyoto, Japan; 13 Department of Diagnostic Pathology, Kyoto University Hospital, Kyoto, Japan; 14 Clinical Research Institute, NHO Kyushu Cancer Center, Fukuoka, Japan

**Keywords:** Anti-HER2 therapy, Lapatinib, Paclitaxel, Phase II study, Trastuzumab

## Abstract

**Background:**

Dual blockade of HER2 promises increased pathological complete response (pCR) rate compared with single blockade in the presence of chemotherapy for HER2-positive (+) primary breast cancer. Many questions remain regarding optimal duration of treatment and combination impact of endocrine therapy for luminal HER2 disease.

**Methods:**

We designed a randomised phase II, five-arm study to evaluate the efficacy and safety of lapatinib and trastuzumab (6 weeks) followed by lapatinib and trastuzumab plus weekly paclitaxel (12 weeks) with/without prolongation of anti-HER2 therapy prior to chemotherapy (18 vs. 6 weeks), and with/without endocrine therapy in patients with HER2+ and/or oestrogen receptor (ER)+ disease. The primary endpoint was comprehensive pCR (CpCR) rate. Among the secondary endpoints, pCR (yT0-isyN0) rate, safety, and clinical response were evaluated.

**Results:**

In total, 215 patients were enrolled; 212 were included in the full analysis set (median age 53.0 years; tumour size = T2, 65%; and tumour spread = N0, 55%). CpCR was achieved in 101 (47.9%) patients and was significantly higher in ER− patients than in ER+ patients (ER− 63.0%, ER+ 36.1%; *P* = 0.0034). pCR with pN0 was achieved in 42.2% of patients (ER− 57.6%, ER+ 30.3%). No significant difference was observed in pCR rate between prolonged exposure groups and standard groups. Better clinical response outcomes were obtained in the prolongation phase of the anti-HER2 therapy. No surplus was detected in pCR rate by adding endocrine treatment. No major safety concern was recognised by prolonging the anti-HER2 treatment or adding endocrine therapy.

**Conclusions:**

This study confirmed the therapeutic impact of lapatinib, trastuzumab, and paclitaxel therapy for each ER− and ER+ subgroup of HER2+ patients. Development of further strategies and tools is required, particularly for luminal HER2 disease.

**Electronic supplementary material:**

The online version of this article (10.1007/s12282-018-0839-7) contains supplementary material, which is available to authorized users.

## Introduction

Trastuzumab has been found to prolong survival and reduce relapse in human epidermal growth factor receptor 2 (HER2)+ patients with advanced and early stage breast cancer when used in combination with other chemotherapy agents and in a neoadjuvant and adjuvant setting [1–4]. In the sequential neoadjuvant and adjuvant settings, the NOAH study compared the treatment with and without trastuzumab in women with HER2+ breast cancer treated with a neoadjuvant chemotherapy regimen [4]. The 3-year event-free survival was higher in the trastuzumab group than the non-trastuzumab group. When comparing the trastuzumab group with the HER2+ chemotherapy alone group, a 41% risk reduction of recurrence, progression, or death by trastuzumab treatment was shown. Similarly, the achievement of higher pathological complete response (pCR) rates with chemotherapy plus trastuzumab has improved survival outcomes in the neoadjuvant setting [5].

Lapatinib, a dual, reversible HER2/epidermal growth factor receptor (HER1) tyrosine kinase inhibitor, both alone and in combination with chemotherapy, has significantly improved progression-free survival in metastatic HER2+ breast cancer patients [6]. Synergistic interactions have been recognised between lapatinib and trastuzumab, owing to their different mechanisms of action and receptor site activity. Studies have shown that combined treatment with lapatinib and trastuzumab was more effective in terms of pCR than trastuzumab alone in patients with HER2+ breast cancer [7–9].

Results of preclinical studies suggest that cross talk between HER2 and oestrogen receptor (ER) signalling pathways in breast cancer contributes to resistance to hormonal therapy [10, 11]. Therefore, it was hypothesised that inhibiting both pathways could be more beneficial than ER or HER2 inhibition alone. In the TAnDEM study, postmenopausal women with HER2/hormone receptor-copositive metastatic breast cancer were treated with anastrozole combined with trastuzumab, without chemotherapy. This combination was found to significantly improve progression-free survival, time to progression, clinical benefit rate, and overall response rate compared with hormone therapy alone [12]. Similarly, in the EGF30008 trial, women with metastatic breast cancer that coexpressed ER and ErbB2 were treated with a combination of lapatinib and letrozole, and achieved significantly increased progression-free survival and clinical benefit rates [13]. In the TBCRC 006 trial, 66 women with locally advanced HER2+ and ER+ breast cancer were treated with neoadjuvant trastuzumab, lapatinib, and letrozole. This approach of targeted therapy without chemotherapy resulted in an overall pCR rate of 27%, and of 21% in ER+ patients [14].

The aim of the present study (JBCRG-16 [Neo-LaTH]) was to evaluate the efficacy and safety of lapatinib and trastuzumab therapy followed by lapatinib and trastuzumab plus weekly paclitaxel in Japanese patients with primary HER2+ breast cancer in a neoadjuvant setting. Additionally, we examined the effects of different periods of lapatinib and trastuzumab therapy (6 vs. 18 weeks) and the effects of add-on endocrine therapy in ER+ patients.

## Patients and methods

### Study design

**Fig. 1:**
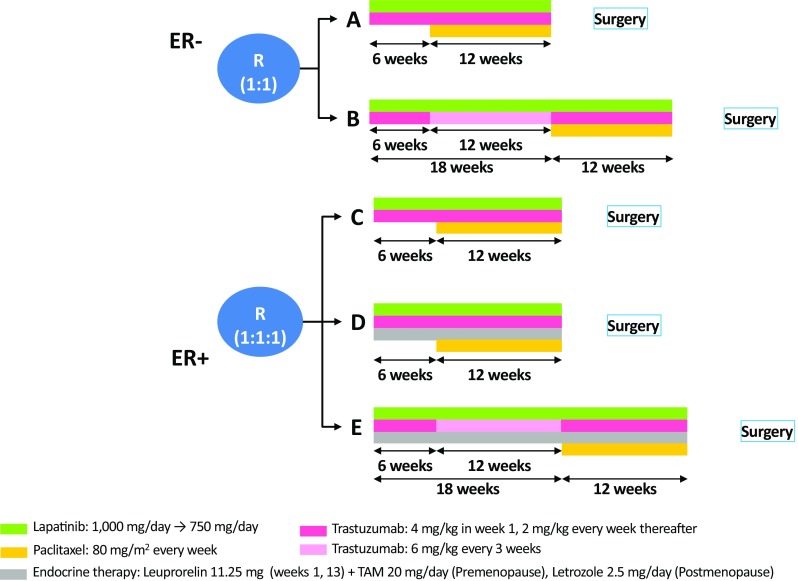
Study design. *ER* oestrogen receptor, *TAM* tamoxifen

This randomised, five-arm, multicentre, open-label phase II trial was conducted between March 2012 and September 2013 in 16 centres in Japan (Fig. 1). This study was registered at http://www.umin.ac.jp/ctr/index-j.htm (UMIN000007576). The study protocol was approved by the ethical review boards of all the participating centres. All the study procedures were conducted in accordance with the latest version of the Declaration of Helsinki and the Good Clinical Practice Guideline.

### Patients

Patients were eligible if they were aged between 20 and 70 years; had a confirmed HER2+ invasive breast cancer by the central laboratory [immunohistochemistry score of 3+ or positive by dual in situ hybridisation (DISH)]; and had primary breast cancer (T1c-3N0-1M0) with a target lesion of ≤ 7 cm. Further details of inclusion and exclusion criteria are described in Supplementary Material S1. All patients provided written informed consent.

### Randomisation

Randomisation, as well as the enrolment of participants and assigning of participants to the trial groups, was performed by the registration centre in Niphix (Tokyo, Japan).

There were five comparative groups (Fig. 1) that contained the following drugs: ER−, (a) lapatinib and trastuzumab followed by the same plus weekly paclitaxel; (b) prolonged lapatinib and trastuzumab followed by the same combined with weekly paclitaxel; ER+, (c) lapatinib and trastuzumab plus weekly paclitaxel, (d) lapatinib and trastuzumab plus weekly paclitaxel and endocrine therapy, and (e) prolonged lapatinib and trastuzumab plus weekly paclitaxel and endocrine therapy. Groups A, C, and D received lapatinib and trastuzumab for 6 weeks; groups B and E, for 18 weeks. All groups received ongoing lapatinib and trastuzumab plus weekly paclitaxel for 12 weeks following the initial lapatinib and trastuzumab period. Some preclinical [15] and clinical [16] studies have suggested that the combination of an aromatase inhibitor and a taxane could be a promising therapeutic option with an additive or synergistic effect. Therefore, after the initial targeted therapy, endocrine therapy was continued during chemotherapy in groups D and E.

### Procedures

Lapatinib was orally administered at 1000 mg/day during the initial lapatinib and trastuzumab period and at 750 mg/day during the lapatinib and trastuzumab plus weekly paclitaxel period. Trastuzumab was administered intravenously at a dose of 4 mg/kg in week 1, and at 2 mg/kg every week thereafter. Paclitaxel was administered by intravenous infusion at a dose of 80 mg/m^2^ every week. Groups D and E received additional endocrine therapy (ET), consisting of 11.25 mg of leuprorelin SR every 12 weeks by subcutaneous depot injection plus 20 mg/day of tamoxifen for premenopausal patients and 2.5 mg/day of letrozole for postmenopausal patients. The procedures are described in detail in Fig. 1 and Supplementary Material S2.

### Outcomes

The primary endpoint was comprehensive pathological complete response (CpCR) rate, including residual ductal carcinoma in situ of the breast. The secondary endpoints were safety profiles and efficacy outcomes, including the clinical overall response rate (ORR), breast conservation rate (BCR), and CpCR plus ypN0. CpCR plus ypN0 was defined as the absence of lymph node metastasis in sentinel node biopsy and/or dissection performed after systemic treatment, even if absence of sentinel lymph node (SLN) metastasis was confirmed before starting therapy.

### Statistical analysis

Among demographic variables, quantitative variables were summarised using mean, median, standard deviation, and maximum and minimum values. The administration of the study drug was summarised as the dose intensity and relative dose intensity descriptively using mean and standard deviation. The adverse event data were categorised by CTCAEv4.0 and analysed. Any patient who received lapatinib treatment was included in the analysis. Regarding the pCR rate, point estimate and two-sided 95% confidence intervals (CIs) were calculated for each between-group difference in pCR rate. Secondary endpoints were analysed using the χ^2^ test or Wilcoxon test based on the type of data. Regarding response rate, point estimates in two-sided 95% CIs were calculated for each group and between-group differences. Between-group comparisons were performed using the χ^2^ test.

We planned a sample size of 40 patients per group (total, 200) to explore three research hypotheses, the details of which are provided in Supplementary Material S2. To evaluate the relationship between the dose of lapatinib during the anti-HER2 therapy and the clinical response, a tail-oriented subpopulation treatment effect pattern plot (STEPP) analysis was performed.

## Results

From 215 patients enrolled, 213 were included in the safety analysis set. One patient, whose tumour was found to be HER2-negative after starting study drug administration, was excluded from the full analysis set (*n* = 212) (Supplementary Material S3).

The characteristics of the patients are summarised in Table 1. Patients had a median age of 53.0 years (range, 26–70 years); 65% were classified as T2 and 55% as N0. Among the 117 N0 patients, 35 (29.9%) underwent pre-systemic therapy SLN biopsy. As a result, 28 patients (80.0%) were SLN-negative. Following successful surgery, 10 patients underwent SLN biopsy again. No axillary metastasis was found in any of these patients.

**Table 1:** Baseline characteristics of the safety analysis set population

	Group A (*n* = 44)	Group B (*n* = 48)	Group C (*n* = 41)	Group D (*n* = 40)	Group E (*n* = 40)	All groups (*n* = 213)
Age (years)						
Median (range)	56.0 (33–69)	56.0 (36–69)	52.0 (32–70)	53.5 (26–66)	49.0 (28–68)	53.0 (26–70)
Menopausal status						
Premenopausal	14 (32)	18 (38)	19 (46)	20 (50)	20 (50)	91 (43)
Postmenopausal	30 (68)	30 (63)	22 (54)	20 (50)	20 (50)	122 (57)
BMI (kg/m^2^)						
Median (range)	21.7 (14.7–32.6)	22.9 (17.3–37.4)	21.8 (14.7–32)	21.5 (14.1–33.7)	21.8 (17.3–35.3)	21.9 (14.1–37.4)
HER2						
IHC 3+	43 (98)	45 (94)	37 (90)	33 (83)	37 (93)	195 (92)
IHC 2 + , FISH+	1 (2)	3 (6)	4 (10)	7 (17)	3 (7)	18 (8)
ER						
ER−	44 (100)	48 (100)	–	–	–	92 (43)
ER+	–	–	41 (100)	40 (100)	40 (100)	121 (57)
T						
T1c	4 (9)	6 (13)	11 (27)	8 (20)	11 (28)	40 (19)
T2	31 (71)	29 (60)	26 (63)	27 (68)	26 (65)	139 (65)
T3	9 (20)	13 (27)	4 (10)	5 (13)	3 (8)	34 (16)
N						
N0	22 (50)	26 (54)	23 (56)	23 (58)	24 (60)	118 (55)
N1	22 (50)	22 (46)	18 (44)	17 (43)	16 (40)	95 (45)
Tumour size (MRI/CT) (mm)						
Median (range)	29 (13–62)	32 (14–70)	24 (11–73)	28 (11–60)	27 (14–61)	28 (11–73)
Breast conservation thought to be possible before starting the trial						
Yes	18 (41)	17 (35)	21 (51)	14 (35)	18 (45)	88 (41)

*BMI* body mass index, *CT* computed tomography, *ER* oestrogen receptor, *FISH* fluorescence in situ hybridisation, *IHC* immunohistochemistry, *MRI* magnetic resonance imaging

Data presented in the table are* n* (%), unless otherwise stated

Regarding the primary study endpoint, CpCR was achieved in 101 (47.9%) patients. This was calculated based on 211 patients in the full analysis set who underwent surgery (one patient elected not to undergo surgery because her lesion had disappeared in response to treatment). CpCR was significantly higher in ER− patients than in ER+ patients (Group A vs. Group C, *P* = 0.0034, Table 2 and Supplementary Material S4). In groups A, B, C, D, and E, CpCR was achieved by 65.9, 60.4, 34.1, 33.3, and 41.0% of patients, respectively. No significant difference was observed in CpCR among the groups with different durations (6 vs. 18 weeks) of lapatinib plus trastuzumab (A vs. B, *P* = 0.59). CpCR in ER+ patients who received add-on endocrine therapy was not significantly greater than that in ER+ patients without endocrine therapy (C vs. D, *P* = 0.94).

**Table 2:** Comprehensive pathological complete response and clinical efficacy

	Comprehensive pathological complete response (%)^a^		Clinical efficacy (%)				
	CpCR (yT0-is)	CpCR (yT0-is) + ypN0	CR in La + T^d^	PR in La + T^e^	ORR in La + T^f^	Additional clinical benefit from paclitaxel	Clinical efficacy at the last time point before surgery^g^
ER−							
A	65.9^b^	61.4^c^	4.5	45.5	50.0	29.5	79.5
B	60.4	54.2	22.9	45.8	68.7	10.4	79.1
ER+							
C	34.1^b^	29.3^c^	4.9	36.6	41.5	41.4	82.9
D	33.3	30.8	0	48.7	48.7	46.2	95.9
E	41	30.8	15	52.5	67.5	22.5	90.0

*CpCR* comprehensive pathological complete response, *CR* complete response, *ER* oestrogen receptor, *La* *+* *T* lapatinib plus trastuzumab, *ns* not significant, *ORR* overall response rate, *PR* partial response

^a^There were no significant differences when comparing A vs. B, C vs. D vs. E, and A + C + D vs. B + E (*P* > 0.05)

^b^Group A vs C: *P* = 0.0034

^c^Group A vs C: *P* = 0.0030

^d^CR rate in La + T period: A vs B, *P* < 0.05; D vs E, *P* < 0.05; C vs D vs E, *P* < 0.05; A + C + D vs B + E, *P* < 0.05

^e^PR rate in La + T period: A vs B, ns; D vs E, ns; C vs D vs E, ns; A + C + D vs B + E, ns

^f^ORR in La + T period: A vs B, ns; D vs E, ns; C vs D vs E, *P* < 0.05; A + C + D vs B + E, *P* < 0.05

^g^There was no significant difference among the five groups in terms of clinical efficacy

The relative dose intensities of lapatinib during the lapatinib and trastuzumab period were over 90%, and during the lapatinib and trastuzumab plus weekly paclitaxel period were around 80% (Supplementary Material S5). Grade ≥ 3 adverse events were observed in 42.3% of patients (Supplementary Material S6); the most common were neutropenia (19%), diarrhoea (12%), skin and subcutaneous disorders, elevated alanine transaminase (5% each), and paronychia (3%). No deaths were reported. There were no significant changes in the mean left ventricular ejection fraction from baseline in any regimen.

CpCR and CpCR + ypN0 were not improved by the extended lapatinib plus trastuzumab or add-on endocrine therapy (Table 2). ORRs evaluated by magnetic resonance imaging (MRI) or computed tomography (CT) were 81.8, 81.3, 85.4, 97.4, and 92.5%; and BCRs were 63.6, 55.3, 70.7, 53.8, and 68.4% (Supplementary Material S4). However, prolongation of lapatinib plus trastuzumab resulted in improved clinical efficacy (Table 2), as shown by clinical response in the lapatinib plus trastuzumab phase evaluated by MRI/CT (ORR and clinical CR). Supplementary Material S7 shows the subpopulation treatment effect pattern plot analysis. The ORR increased linearly with increasing lapatinib dose in the lapatinib plus trastuzumab period especially in the ER+ cohort.

A similar tendency was observed in quasi-pCR [QpCR (CpCR + near pCR)]. For ER+ patients, quasi-pCR tended to improve with increasing lapatinib dose and add-on ET (Supplementary Material S8). Clinical efficacy at the last time point before surgery (i.e., at the end of weekly paclitaxel add-on period following lapatinib and trastuzumab period) is shown in Table 2. There was no significant difference among the five groups in terms of clinical efficacy. In groups B and E, the benefit from the add-on weekly paclitaxel tended to be small.

**Fig. 2:**
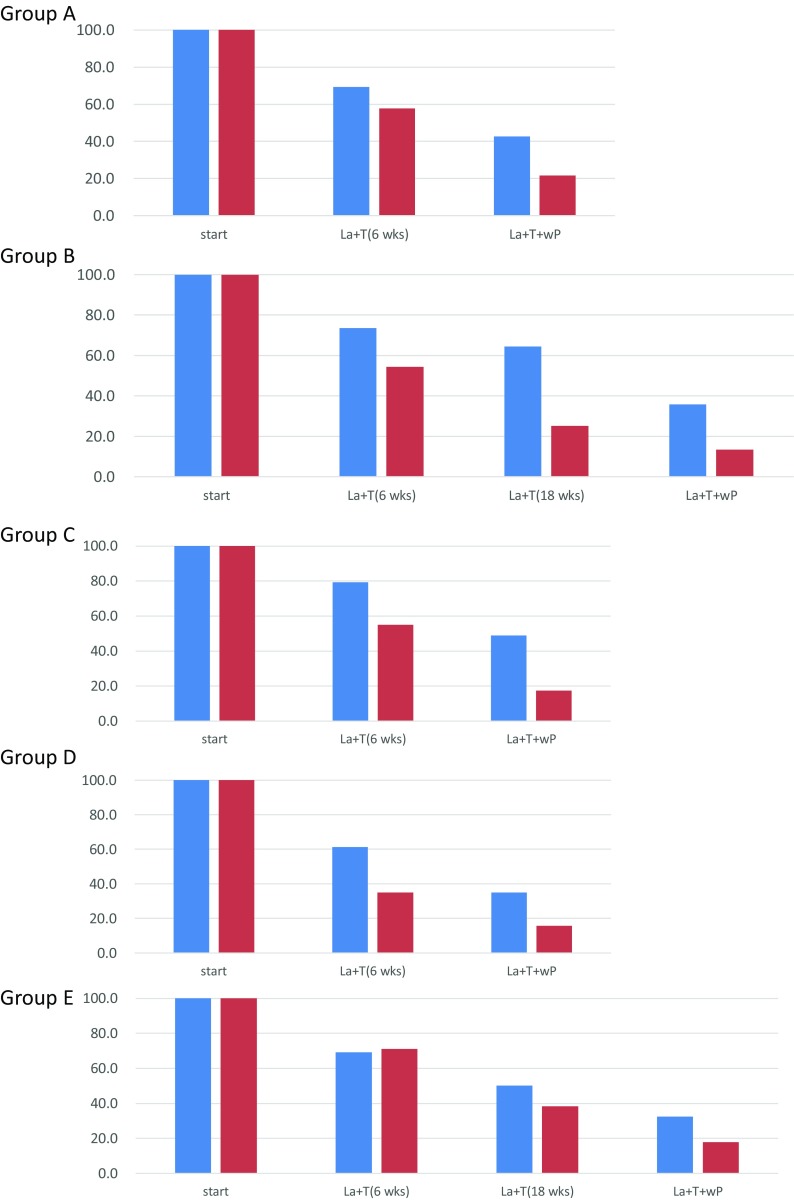
Percent change in maximum tumour size from pre-treatment to end of treatment measured by MRI/CT during the time course and relationship between pathological response per group. The vertical axis shows the percent change in maximum tumour size from pre-treatment to end of treatment measured by MRI or CT. Considering that tumour sizes varied among patients, the mean maximum tumour size was set at 100% at the study start. Then, the percent change in maximum tumour size was calculated at the end of treatment. The percent change values shown in the graph represent the percentage of the tumour size remaining at the end of treatment. The horizontal axis is the timing of the examination course. Red column: patients who achieved pathological complete response (pCR); blue column: patients with residual invasive disease (non-pCR). *MRI* magnetic resonance imaging, *CT* computed tomography, *La* lapatinib, *T* trastuzumab, *wP* weekly paclitaxel

From the experimental analyses regarding the percent change in maximum tumour size from pre-treatment to end of treatment measured by MRI/CT during the time course and relationship between pathological response (Fig. 2), among ER− patients, the prolongation of the lapatinib plus trastuzumab phase seemed to be important for achieving pCR. Conversely, among ER+ patients, pCR was mainly influenced by the reduction effect through the addition of paclitaxel. This tendency was observed especially in groups C and D. In Group B, at 18 weeks of lapatinib plus trastuzumab therapy, the percent change in maximum tumour size was 25.1% (95% CI 13.5–36.8%) in patients with pCR and 64.5% (95% CI 45.7–83.4%) in patients without pCR, indicating a significant reduction in the patients with pCR. In the hormone receptor-positive groups C and D, the percent change in maximum tumour size at the end of the lapatinib plus trastuzumab plus weekly paclitaxel therapy (at the end of all the cycles) was significantly different between patients with and without pCR. In Group C, the percent change in maximum tumour size was 17.4% (95% CI 4.4–30.5%) in patients with pCR and 49.0% (95% CI 37.1–60.9%) in patients without pCR. In Group D, the ratio was 15.7% (95% CI 2.2–29.3%) in patients with pCR and 34.9% (95% CI 25.2–44.6%) in patients without pCR.

Supplementary Material S9 shows the changes in tumour diameter from baseline to the end of treatment. Among ER− patients, those who responded to treatment early in the lapatinib plus trastuzumab phase tended to achieve pCR. Among ER+ patients, there was no significant correlation between clinical efficacy and pathological efficacy.

## Discussion

In the Neo-LaTH study, the efficacy and safety of lapatinib and trastuzumab therapy followed by lapatinib and trastuzumab plus weekly paclitaxel in primary HER2+ breast cancer patients were equivalent to that in two previous studies [7, 8]. Regarding the effect of the extension of lapatinib plus trastuzumab treatment period from 6 to 18 weeks and addition of endocrine therapy, the pCR rates (primary endpoint) were not improved by the extended treatment. Although the primary endpoint did not match the expected result, the secondary clinical efficacy indicates that in ER− patients finally achieving pCR, combined HER2 blockade therapy alone, which was given before the initiation of the weekly paclitaxel treatment, tended to induce a greater reduction due to the prolongation of the therapy from 6 to 18 weeks. This suggests that in ER− patients who respond to combined HER2 blockade therapy, the prolongation of the treatment period can lead to the avoidance of the future use of cytotoxic anti-cancer drugs. The comparison of Groups D and E with combined hormone therapy showed that among ER+ patients, the proportion of patients with complete response or partial response tended to be greater when the treatment period was prolonged to 18 weeks. However, the difference was not as great as that observed in ER− patients. Our findings are in agreement with previous chemotherapy-free regimens (the NeoSPHERE [17] and the NeoALTTO [7] studies). Thus, the results of the chemotherapy-free phase of Neo-LaTH support the concept that dual HER2 blockage can lead to decreased length of chemotherapy regimens, and that a proportion of HER2+ tumours could potentially be eradicated with chemotherapy-free regimens.

Changes in tumour diameter tended to be larger after add-on endocrine therapy and extended treatment with lapatinib and trastuzumab in ER+ patients in the present study. Similarly, 40% of patients in the TBCRC 006 study achieved pCR by 12 weeks of dual HER2 blockage with lapatinib plus trastuzumab despite having large initial tumour size [14]. Together, these results suggest that the changes in tumour diameter increase in proportion to the extended period of molecularly targeted drug therapy. Overall clinical response rates were also significantly higher in the 18-week group rather than the 6-week group. Our findings are useful for the future exploration of chemotherapy-free anti-HER2 regimens for women who cannot receive cytotoxic drugs.

We hypothesised that the addition of endocrine therapy may increase the pCR rate in ER+ luminal HER2 subtype. Regimen D showed high overall clinical response rate (97.4%) with 31.6% of clinical CR. In the regimen-E group, it was also as high as 92.5% with 37.5% of clinical CR, indicating that the endocrine therapy combination would be able to provide high levels of clinical response. However, pCR in ER+ patients who received add-on endocrine therapy was not significantly greater than that in ER+ patients without endocrine therapy. These results were unexpected since improved pCR rates had been achieved previously in the TBCRC 006 study of women with locally advanced HER2+ and ER+ breast cancer who were treated with neoadjuvant trastuzumab, lapatinib, and letrozole [14]. To increase the pCR rate against luminal HER2 disease, further strategies need to be developed.

With regard to toxicity, the frequencies of grade 3/4 adverse events in the Neo-LaTH study were similar to those observed in other studies. The relative dose intensity of lapatinib was also similar to that in previous results. From the STEPP analysis, a relationship was revealed between the administration dose of lapatinib during the anti-HER2 therapy phase and the clinical response.

The strength of the present study is the novelty of the study design, which allowed the prolonged duration of dual HER2 blockage with lapatinib plus trastuzumab in the neoadjuvant setting. This study had several potential limitations, in particular those inherent to phase II open-label studies. However, current outcomes may be informative for future studies, and correlative translational research is ongoing to analyse molecular mechanisms of tumour responses and therapeutic resistance. Another limitation is the small sample size; however, this was determined on a statistical basis, and it is important to verify a hypothesis in a small sample size study.

In conclusion, the findings of the present study confirmed the efficacy and safety of lapatinib and trastuzumab followed by lapatinib plus trastuzumab plus weekly paclitaxel for the treatment of HER2+ invasive breast cancer in Japanese patients. These results are relevant for clinicians considering the optimal duration of preoperative anti-HER2 treatment for HER2+ disease and the combination with endocrine therapy for luminal HER2 subtype. Further analysis of disease-free survival/overall survival in conjunction with biomarker studies is necessary to explore the therapeutic impact.

## Electronic supplementary material

Below is the link to the electronic supplementary material.

Supplementary material 1 (PPTX 841 kb)

Supplementary material 2 (DOCX 40 kb)

Supplementary material 3 (DOCX 49 kb)
