# Supplementary material for: Efficacy and safety of trastuzumab, lapatinib, and paclitaxel neoadjuvant treatment with or without prolonged exposure to anti-HER2 therapy, and with or without hormone therapy for HER2-positive primary breast cancer: a randomised, five-arm, multicentre, open-label phase II trial
Source: Breast Cancer. 2018 Feb 14;25(4):407–15. doi: 10.1007/s12282-018-0839-7 (PMC5996004; doi:10.1007/s12282-018-0839-7)
Supplement: Supplementary file 1 — Supplementary material 1 (PPTX 841 kb) [file 12282_2018_839_MOESM1_ESM.pptx]

## Slide 1
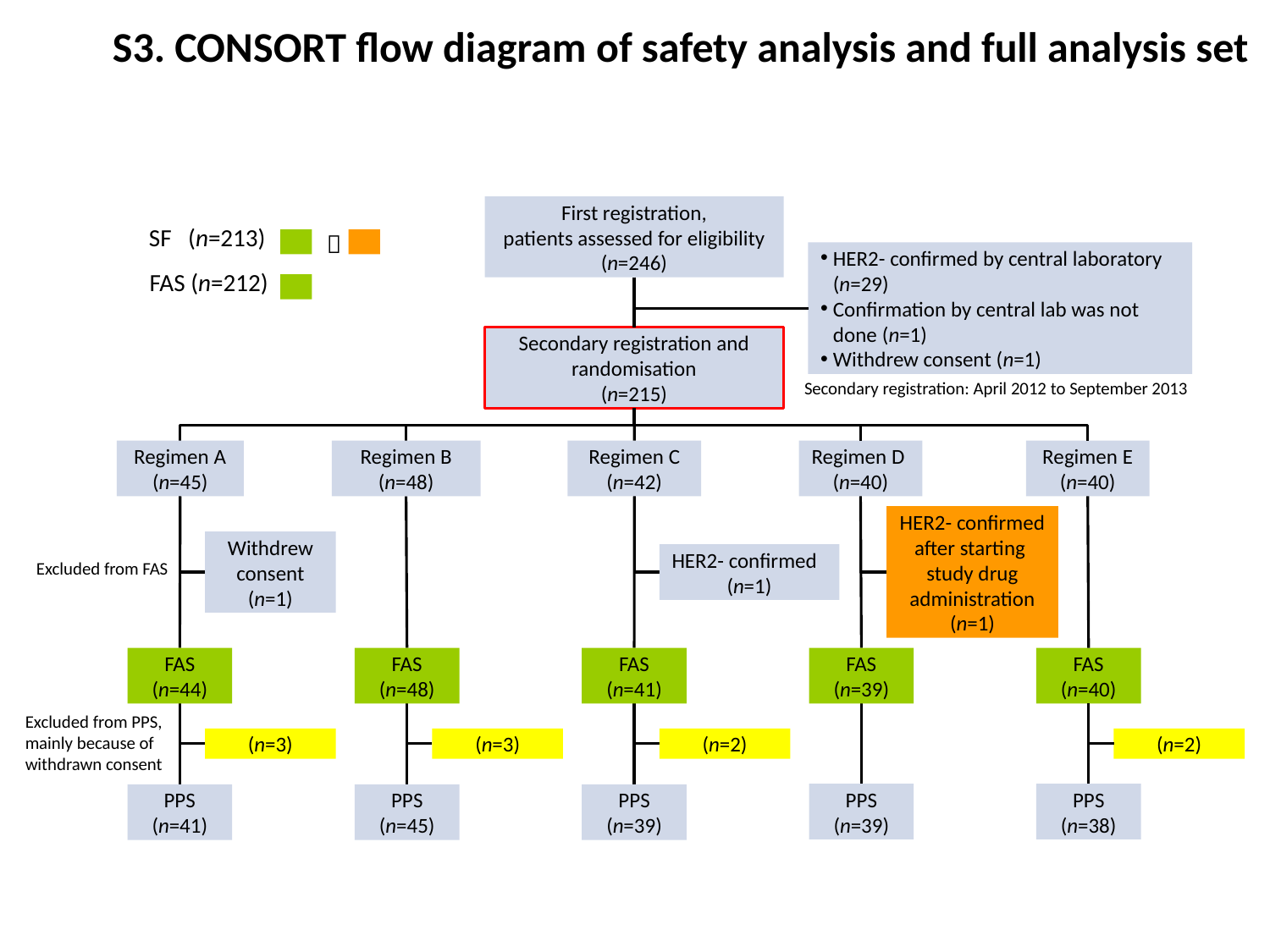

S3. CONSORT flow diagram of safety analysis and full analysis set
First registration,
patients assessed for eligibility
(n=246)
SF (n=213)
＋
FAS (n=212)
HER2- confirmed by central laboratory (n=29)
Confirmation by central lab was not done (n=1)
Withdrew consent (n=1)
Secondary registration and randomisation
(n=215)
Secondary registration: April 2012 to September 2013
Regimen A
(n=45)
Regimen B
(n=48)
Regimen C
(n=42)
Regimen D
(n=40)
Regimen E
(n=40)
HER2- confirmed
after starting
study drug
administration
(n=1)
Withdrew consent
(n=1)
HER2- confirmed
(n=1)
Excluded from FAS
FAS
(n=44)
FAS
(n=48)
FAS
(n=41)
FAS
(n=39)
FAS
(n=40)
Excluded from PPS,
mainly because of
withdrawn consent
(n=3)
(n=2)
(n=2)
(n=3)
PPS
(n=39)
PPS
(n=38)
PPS
(n=41)
PPS
(n=45)
PPS
(n=39)

## Slide 2
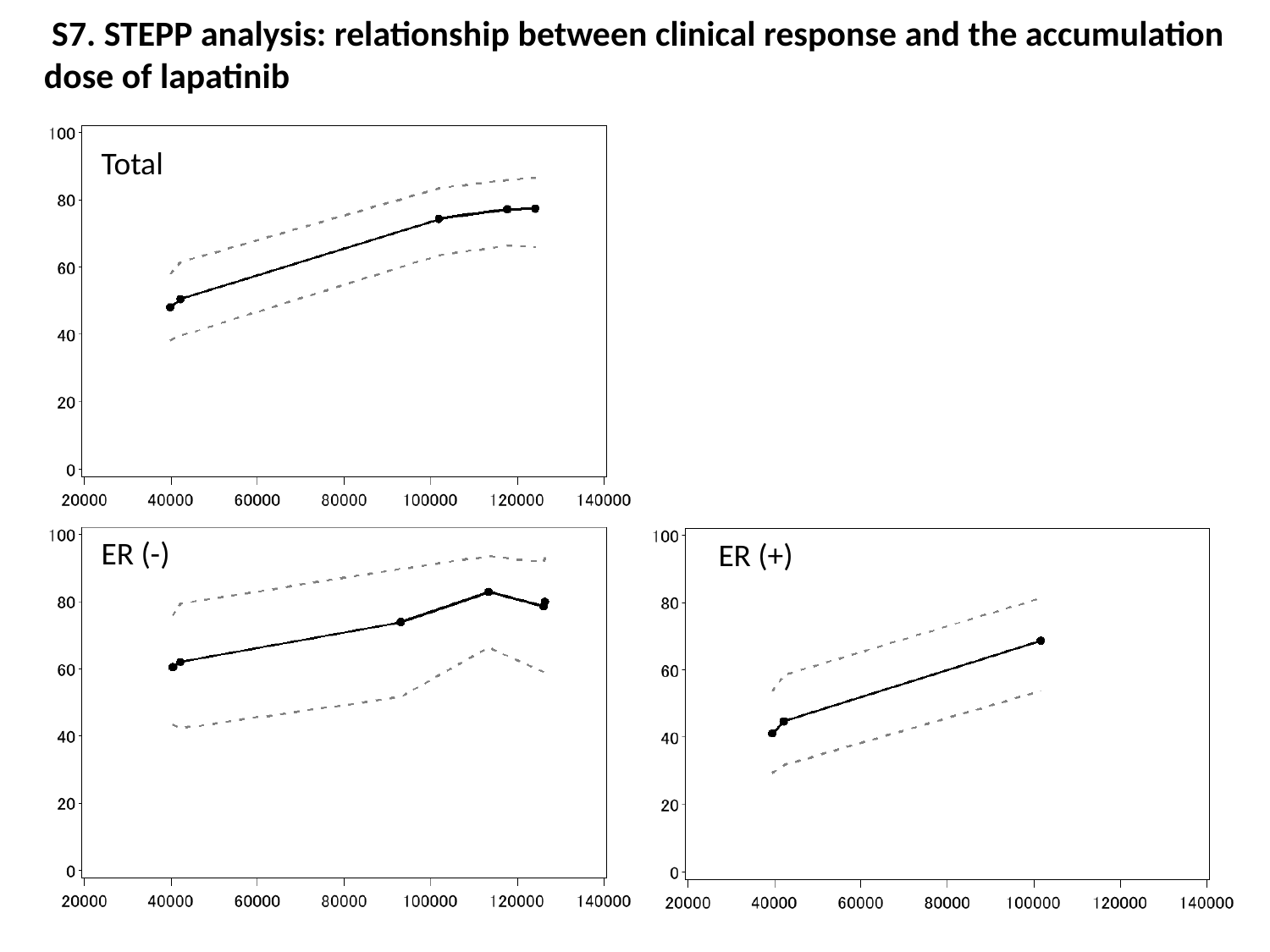

S7. STEPP analysis: relationship between clinical response and the accumulation dose of lapatinib
Total
ER (-)
ER (+)

## Slide 3
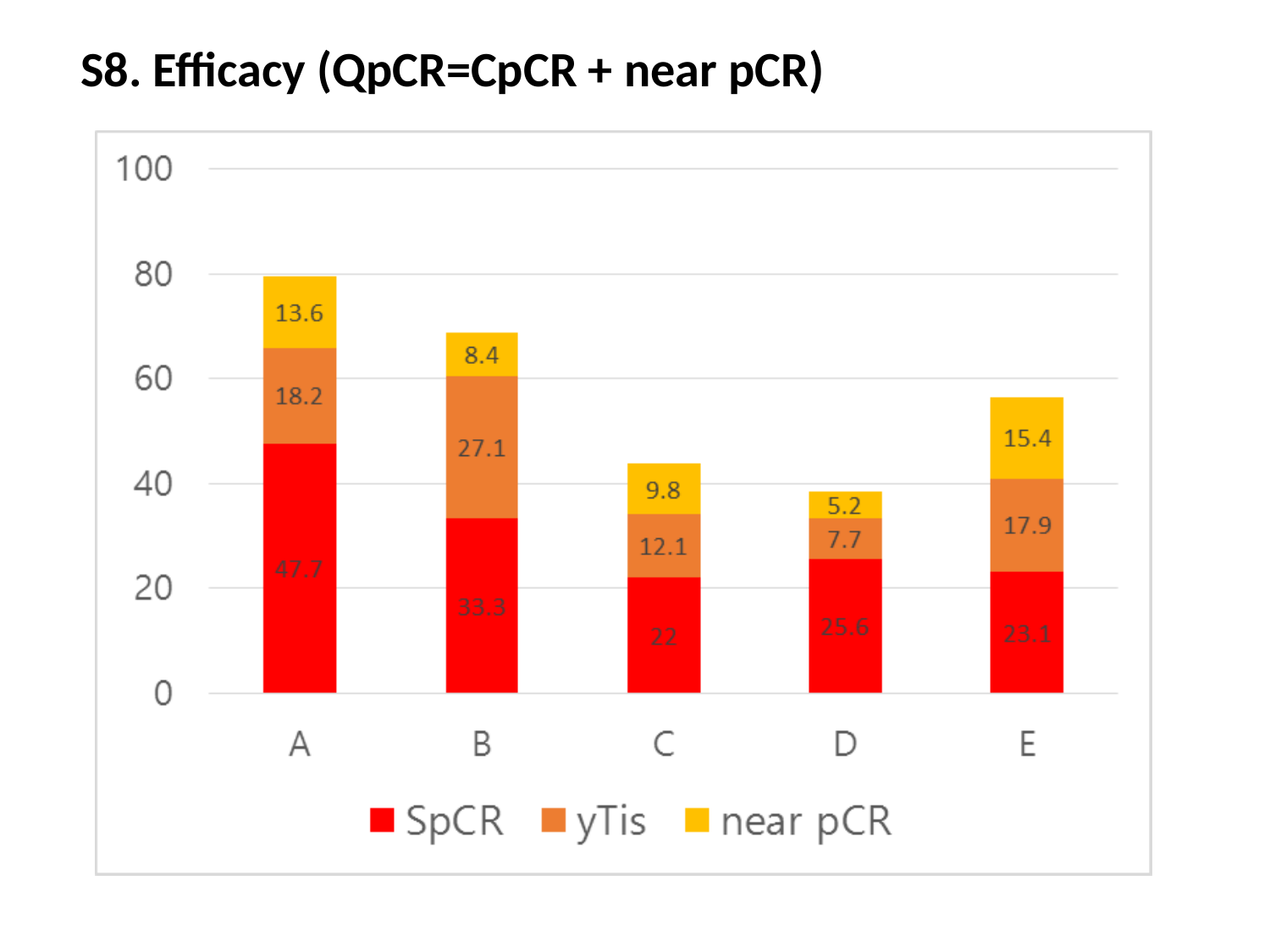

S8. Efficacy (QpCR=CpCR + near pCR)

## Slide 4
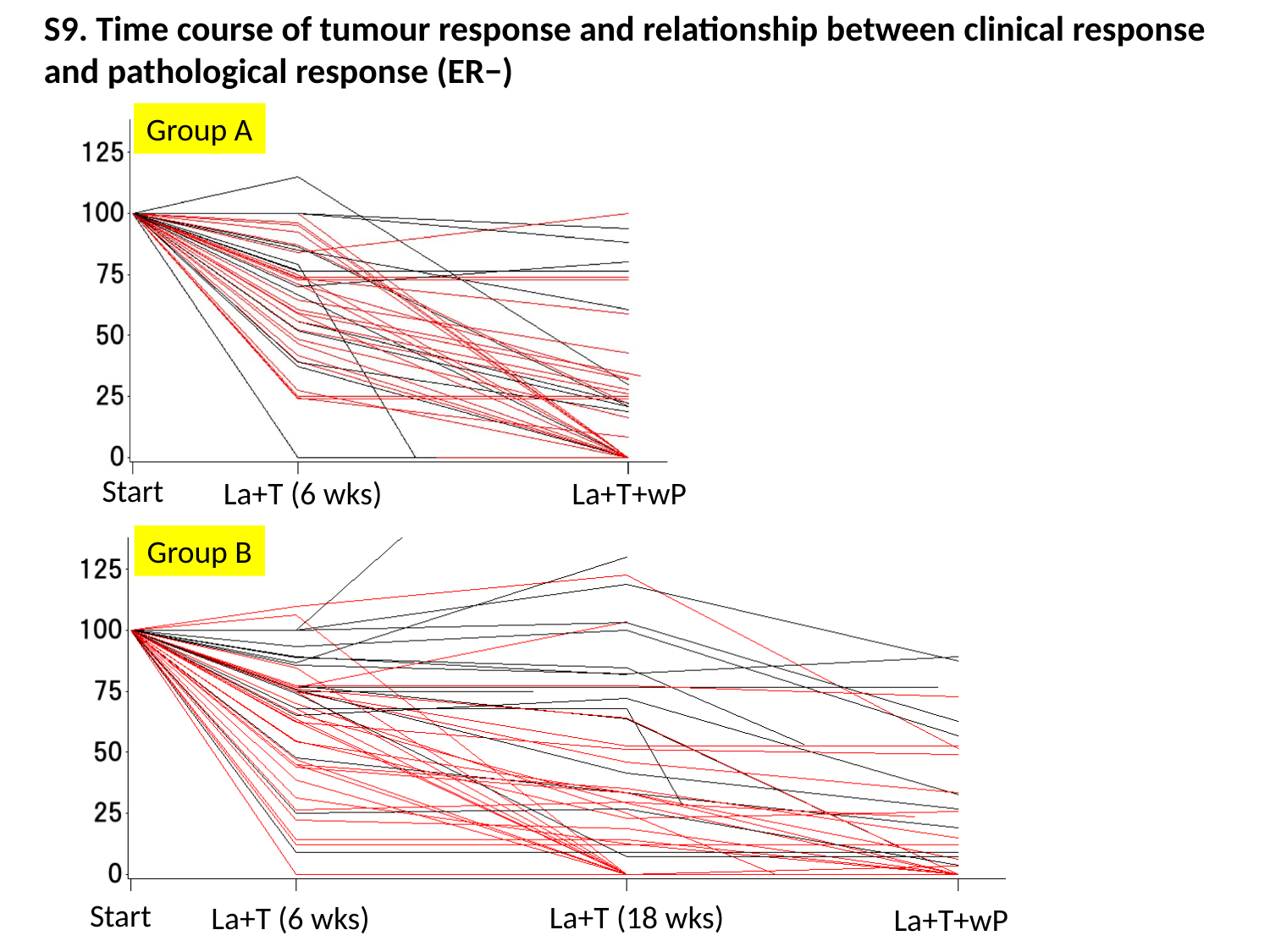

S9. Time course of tumour response and relationship between clinical response and pathological response (ER−)
Group A
Start
La+T (6 wks)
La+T+wP
Group B
Start
La+T (18 wks)
La+T (6 wks)
La+T+wP

## Slide 5
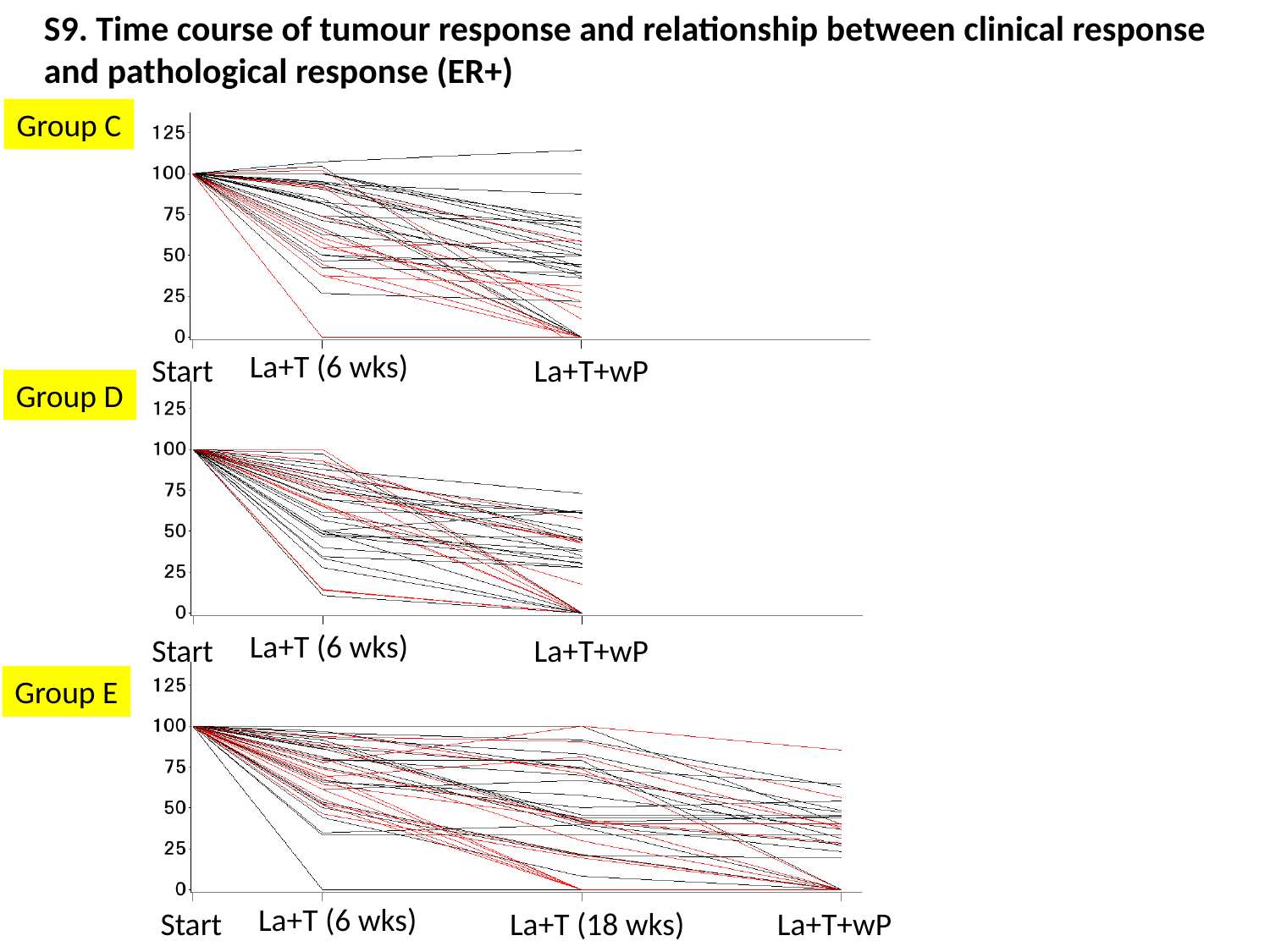

S9. Time course of tumour response and relationship between clinical response and pathological response (ER+)
Group C
La+T (6 wks)
Start
La+T+wP
Group D
La+T (6 wks)
Start
La+T+wP
Group E
La+T (6 wks)
Start
La+T (18 wks)
La+T+wP
