# Supplementary material for: Efficacy and safety of trastuzumab, lapatinib, and paclitaxel neoadjuvant treatment with or without prolonged exposure to anti-HER2 therapy, and with or without hormone therapy for HER2-positive primary breast cancer: a randomised, five-arm, multicentre, open-label phase II trial
Source: Breast Cancer. 2018 Feb 14;25(4):407–15. doi: 10.1007/s12282-018-0839-7 (PMC5996004; doi:10.1007/s12282-018-0839-7)
Supplement: Supplementary file 2 — Supplementary material 2 (DOCX 40 kb) [file 12282_2018_839_MOESM2_ESM.docx]

**Supplementary material**

**S1. Study eligibility criteria**

| **Inclusion criteria** | **Exclusion criteria** |
| --- | --- |
| 1.First key inclusion criteria  (1) Age between 20 and 70 years  (2) Female patients with primary breast cancer diagnosed as invasive cancer by needle biopsy or tissue biopsy  (3) Resectable primary breast cancer (T1c-3N0-1M0) with a tumour size <7 cm in diameter (multiple ipsilateral breast cancer was eligible when at least one lesion met the eligibility criteria. However, each lesion had to be histologically evaluated)  (4) Invasive component of the primary tumour confirmed as HER2-positive (IHC 3+ or FISH+)  (5) Oestrogen receptor and progesterone receptor statuses confirmed by IHC  (6) No previous therapy for breast cancer  (7) Patient confirmed as suitable for primary systemic therapy  (8) Imaging evaluation of the primary lesion could be performed at baseline and after the end of study treatment using the same modality (either CT scan, MRI, or ultrasonography)  (9) Written informed consent  2. Secondary criteria  (1) HER2-positive invasive ductal carcinoma confirmed by the pathological central review  (2) ECOG performance status 0–1  (3) Laboratory test results met the following criteria (within 14 days before registration): neutrophil count: ≥1,500/mm3; haemoglobin: ≥9.0 g/dL; platelet count: ≥100,000/mm3; AST and ALT: ≤2.5× upper limit of normal (ULN) established at site; ALP: ≤2.5× ULN; total bilirubin: ≤1.5× ULN; serum creatinine: ≤1.5× ULN  (3) Baseline left ventricular ejection fraction ≥50% measured by echocardiography or MUGA scan  (4) No QTc prolongation by electrocardiography (QTc: ≤470 ms)  (5) No interstitial pneumonia or pulmonary fibrosis diagnosed by chest CT scan  (6) Negative determination of HBsAg in hepatitis B screening  (7) If a patient’s postmenopausal status could not be confirmed, her pregnancy test must have been negative (urinary or serum human chorionic gonadotropin negative) (excluding ovariectomised or hysterectomised patients) | (1) History of drug hypersensitivity that was relevant to the study treatment (i.e., past history of immediate or delayed hypersensitivity reaction to compounds chemically similar to lapatinib and its excipients)  (2) Uncontrolled concurrent disease  (3) Active infection, or pyrexia that indicates suspected infection  (4) Symptoms of varicella  (5) Pleural or pericardial effusion requiring treatment  (6) Past gastric or small bowel resection, or malabsorption or gastrointestinal dysfunction, except for ulcerative colitis  (7) Use of concomitant medication (e.g., CYP3A4 inhibitors/inducers) or non-drug therapy prohibited  (8) Current chronic use of systemic corticosteroids; in ER+ patients, current treatment with any drug product containing oestrogen or any selective oestrogen receptor modulator |

Abbreviations: ALT, alanine transaminase; AST, aspartate transaminase; CT, computed tomography; ECOG, Eastern Cooperative Oncology Group; FISH, fluorescence in situ hybridisation; IHC, immunohistochemistry; MRI, magnetic resonance imaging; MUGA, multigated acquisition

**S2. Additional details of the procedures and sample size calculations**

*Procedures*

HER2 and ER status were confirmed by the central laboratory. Immunohistochemical stains were performed using the Ventana BenchMark ULTRA and iView Detection Kit (Roche Diagnostics Corp., Tucson, USA). Primary antibodies used were HER2 (Ventana PATHWAY anti-HER2/neu [4B5] rabbit monoclonal antibody) and ER (Ventana CONFIRM anti-ER [SP1]). HER2 overexpression was identified if a strong complete membranous stain was observed in more than 30% of cancer cells immunohistochemically (Roche monoclonal antibody 4B5) or HER2/CEP17 > 2.0 in DISH (Ventana INFORM dual ISH kit). The percentage of ER+ cancer cells was counted in a hot spot of 500 cancer cells, and the cut-off of ER+ cells was more than 1%. Resected specimens were also examined by the central assessment to confirm the pCR category according to the status of residual non-invasive and/or invasive cancer cells [1].

*Sample size calculations*

First, in ER− patients, we investigated if improvement in pCR rate was expected as a result of extension of the lapatinib plus trastuzumab treatment period from 6 to 18 weeks. The pCR rate with standard treatment on arm A was estimated to be approximately 60% [2]. When evaluating if there was a 15% improvement in the pCR rate using a one-sided 20% α error, a detection power of 70% was secured with 37 patients per group. Second, in patients with ER+ tumours, we investigated if the addition of hormone therapy resulted in an improvement in pCR rate. Then, we investigated if extension of the lapatinib plus trastuzumab treatment period, in addition to hormone therapy, resulted in further improvement in the pCR rate. The pCR rate with standard treatment on arm C was estimated to be approximately 40% [2]. When evaluating if there was a 15% improvement in pCR rate by 15% as a result of the addition of hormone therapy using a one-sided 20% α error, a detection power of 70% was secured with 42 patients per group. When evaluating if there was a 15% further improvement in the pCR rate under the same condition, a detection power of 70% was secured with 39 patients per group. In this study, the target sample size was determined to be 80 patients in the ER− group, 120 patients in the ER+ group, and a total of 200 patients in the whole study.

**References**

1. Kuroi K, Toi M, Tsuda H et al. Issues in the assessment of the pathologic effect of primary systemic therapy for breast cancer. Breast Cancer 2006; 13: 38-48.

2. Baselga J, Bradbury I, Eidtmann H et al. Lapatinib with trastuzumab for HER2-positive early breast cancer (NeoALTTO): a randomised, open-label, multicentre, phase 3 trial. Lancet 2012; 379: 633-640.

**S3. CONSORT flow diagram of safety analysis and full analysis set**

Abbreviations: FAS, full analysis set; SF, safety analysis set; PPS, per-protocol set

**S4. Efficacy**

**S5. Dose intensity and relative dose intensity of lapatinib**

**S6. Frequencies of grade 3/4 adverse events (≥ 5 events)**

**S7. STEPP analysis: relationship between clinical response and the accumulation dose of lapatinib**

The vertical axis shows the overall clinical response rate after lapatinib and trastuzumab combination therapy. The horizontal axis shows the average of accumulated lapatinib dose (mg). The dotted line shows the 95% confidence interval.

Abbreviations: ER, oestrogen receptor; STEPP, subpopulation treatment effect pattern plot

**S8. Efficacy (QpCR=CpCR+near pCR)**

Abbreviations: CpCR, comprehensive pathological complete response; pCR, pathological complete response; QpCR, quasi-pCR

**S9. Time course of tumour response and relationship between clinical response and pathological response (ER− and ER+)**

The vertical axis shows the tumour volume ratio measured by magnetic resonance imaging or computed tomography. The horizontal axis shows the timing of examination. The red line shows patients who achieved pCR. The black line shows patients with residual invasive disease (non-pCR)

Abbreviations: ER, oestrogen receptor; La, lapatinib; pCR, pathological complete response; T, trastuzumab; wP, weekly paclitaxel

**S10. List of participating centres**

National Hospital Organization Osaka National Hospital; Gunma Prefectural Cancer Center; Chiba Cancer Center; Saitama Cancer Center; University of Tsukuba Hospital; Toranomon Hospital; Tokyo Metropolitan Cancer and Infectious Diseases Center Komagome Hospital; Kanagawa Cancer Center; Aichi Cancer Center Hospital; Hiroshima City Hiroshima Citizens Hospital; National Hospital Organization Shikoku Cancer Center; National Hospital Organization Kyushu Cancer Center; Nihon University Itabashi Hospital; National Hospital Organization Kure Medical Center and Chugoku Cancer Center; Kyoto University Hospital.
