# Supplementary material for: Efficacy and safety of trastuzumab, lapatinib, and paclitaxel neoadjuvant treatment with or without prolonged exposure to anti-HER2 therapy, and with or without hormone therapy for HER2-positive primary breast cancer: a randomised, five-arm, multicentre, open-label phase II trial
Source: Breast Cancer. 2018 Feb 14;25(4):407–15. doi: 10.1007/s12282-018-0839-7 (PMC5996004; doi:10.1007/s12282-018-0839-7)
Supplement: Supplementary file 3 — Supplementary material 3 (DOCX 49 kb) [file 12282_2018_839_MOESM3_ESM.docx]

**S4. Efficacy**

|  | **Regimen A**  **(*n*=44)** | **Regimen B**  **(*n*=48)** | **Regimen C**  **(*n*=41)** | **Regimen D**  **(*n*=39)** | **Regimen E**  **(*n*=39)^a^** | **Regimens A+C (*n*=85)**  **LaT 6w + LaTP 12w** | **Regimens A+C+D (*n*=124)**  **LaT 6w + LaTP 12w**  **(D: +Hormone therapy)** | **Regimens B+E (*n*=87)**  **LaT 18w + LaTP 12w**  **(E: +Hormone therapy)** | ***P*-value^b^** |
| --- | --- | --- | --- | --- | --- | --- | --- | --- | --- |
| **CpCR (*n*, %)** | 29 (65.9) | 29 (60.4) | 14 (34.1) | 13 (33.3) | 16 (41.0) | 43 (50.6) | 56 (45.2) | 45 (51.7) | A vs. C:  *P*=0.0034 |
| **CpCR +ypN0 (*n*, %)** | 27 (61.4) | 26 (54.2) | 12 (29.3) | 12 (30.8) | 12 (30.8) | 39 (45.9) | 51 (41.1) | 38 (43.7) | A vs. C:  *P*=0.0030 |
| **Overall response by MRI or CT (*n*, %)** | 36 (81.8) | 39 (81.3) | 35 (85.4) | 37 (97.4) | 37 (92.5) | 71 (83.5) | 108 (87.8) | 76 (86.4) |  |
| **CR** | 17 (38.6) | 18 (37.5) | 10 (24.4) | 12 (31.6) | 15 (37.5) | 27 (31.8) | 39 (31.7) | 33 (37.5) |  |
| **PR** | 19 (43.2) | 21 (43.8) | 25 (61.0) | 25 (65.8) | 22 (55.0) | 44 (51.8) | 69 (56.1) | 43 (48.9) |  |
| **SD** | 7 (15.9) | 9 (18.8) | 5 (12.2) | 1 (2.6) | 1 (2.5) | 12 (14.1) | 13 (10.6) | 10 (11.4) |  |
| **PD** | 0 | 0 | 0 | 0 | 1 (2.5) | 0 | 0 | 1 (1.1) |  |
| **NE** | 1 (2.3) | 0 | 1 (2.4) | 0 | 1 (2.5) | 2 (2.4) | 2 (1.6) | 1 (1.1) |  |
| **Breast conserving surgery, *n* (%) in all patients**  **Cut-end negative (free margin), *n* (%)** | 28 (63.6)  26 (59.1) | 26 (55.3)  24 (51.1) | 29 (70.7)  25 (61.0) | 21 (53.8)  16 (41.0) | 26 (68.4)  24 (63.2) | 57 (67.1)  51 (60.0) | 78 (62.9)  67 (54.0) | 52 (61.2)  48 (56.5) |  |
| **Breast conserving surgery, *n* (%) in patients planned to undergo mastectomy before the trial**  **Cut-end negative (free margin), *n* (%)** | 12 (50.0)  10 (41.7) | 11 (36.7)  9 (30.0) | 10 (55.6)  8 (44.4) | 7 (31.8)  4 (18.2) | 11 (64.7)  9 (52.9) | 22 (52.4)  18 (42.9) | 29 (45.3)  22 (34.4) | 22 (46.8)  18 (38.3) |  |

^a^One patient did not wish to undergo surgery because her lesion had disappeared in response to treatment.

^b^There were no significant differences (*P*>0.05) in comparisons of CpCR between A vs. B (*P*=0.5856), C vs. D (*P*=0.9387), D vs. E, and A+C+D vs. B+E.

^c^There were no significant differences (*P*>0.05) in comparisons of CpCR between A+C+D vs. B+E.

Abbreviations: PR, partial response; SD, stable disease; PD, progressive disease; NE, non-evaluable; CI, confidence interval; CpCR, comprehensive pathological complete response; CR, complete response; CT, computed tomography; MRI, magnetic resonance imaging.

**S5. Dose intensity and relative dose intensity of lapatinib**

|  | **HR-** | | | | | | | **HR+** | | | | | | | | | |
| --- | --- | --- | --- | --- | --- | --- | --- | --- | --- | --- | --- | --- | --- | --- | --- | --- | --- |
|  | **Regimen A**  **(*n*=44)** | | | | **Regimen B**  **(*n*=48)** | | | **Regimen C**  **(*n*=41)** | | | | **Regimen D**  **(*n*=40)** | | | **Regimen E**  **(*n*=40)** | | |
|  | | **LaT** | **LaT+wP** | **Overall** | **LaT** | **LaT+wP** | **Overall** | **LaT** | **LaT+wP** | **Overall** | **LaT** | | **LaT+wP** | **Overall** | **LaT** | **LaT+wP** | **Overall** |
| *n* | | 44 | 43 | 44 | 48 | 41 | 48 | 41 | 38 | 41 | 40 | | 38 | 40 | 40 | 36 | 40 |
| Dose intensity (mg/day) | | 977.2  63.5 | 680.0  133.0 | 807.2  54.6 | 956.0  53.1 | 620.8  262.6 | 878.8  80.9 | 982.8  53.1 | 657.8  199.8 | 818.0  69.8 | 981.4  70.1 | | 666.7  167.8 | 807.2  67.3 | 959.2  85.9 | 639.7  223.9 | 874.2  75.6 |
| Relative dose intensity (%) | | 97.1  7.2 | 81.7  27.3 | 87.8  17.0 | 91.7  17.4 | 72.7  38.8 | 85.4  22.5 | 94.9  15.5 | 80.5  30.4 | 86.2  23.3 | 96.0  12.3 | | 86.0  24.0 | 90.0  17.2 | 90.4  21.1 | 78.2  34.2 | 86.3  24.1 |

Data in the table are presented as mean and standard deviation. Planned ideal total doses of lapatinib were 105,000 mg (regimens A, C, and D) and 189,000 mg (regimens B and E).

Abbreviations: LaT, lapatinib plus trastuzumab; wP, weekly paclitaxel

**S6. Frequencies of grade 3/4 adverse events (≥ 5 events)**

|  | **Group A**  **(*n*=44)** | | **Group B**  **(*n*=48)** | | **Group C**  **(*n*=41)** | | **Group D**  **(*n*=40)** | | **Group E**  **(*n*=40)** | | **All groups**  **(*n*=213)** | |
| --- | --- | --- | --- | --- | --- | --- | --- | --- | --- | --- | --- | --- |
|  | *N* (%) | Events | *N* (%) | Events | *N* (%) | Events | *N* (%) | Events | *N* (%) | Events | *N* (%) | Events |
| All events | 20 (45.5) | 35 | 18 (37.5) | 33 | 15 (36.6) | 29 | 19 (47.5) | 36 | 18 (45.0) | 29 | 90 (42.3) | 162 |
| Neutropenia | 11 (25.0) | 13 | 9 (18.8) | 9 | 8 (19.5) | 9 | 8 (20.0) | 14 | 5 (12.5) | 8 | 41 (19.2) | 53 |
| Diarrhoea | 5 (11.4) | 5 | 3 (6.3) | 3 | 5 (12.2) | 5 | 8 (20.0) | 8 | 4 (10.0) | 4 | 25 (11.7) | 25 |
| Skin rash | 4 (9.1) | 4 | 2 (4.2) | 2 | 2 (4.9) | 2 | 1 (2.5) | 1 | 2 (5.0) | 2 | 11 (5.2) | 11 |
| ALT increased | 3 (6.8) | 3 | 3 (6.3) | 3 | 3 (7.3) | 3 | 2 (5.0) | 2 |  |  | 11 (5.2) | 11 |
| Paronychia |  |  | 3 (6.3) | 3 |  |  | 1 (2.5) | 1 | 3 (7.5) | 3 | 7 (3.3) | 7 |
| Peripheral motor neuropathy |  |  | 1 (2.1) | 1 | 1 (2.4) | 1 | 2 (5.0) | 2 | 2 (5.0) | 2 | 6 (2.8) | 6 |
| Fatigue |  |  |  |  | 1 (2.4) | 2 | 1 (2.5) | 1 | 3 (7.5) | 3 | 5 (2.3) | 6 |
| Anorexia | 3 (6.8) | 3 | 1 (2.1) | 1 | 1 (2.4) | 1 |  |  |  |  | 5 (2.3) | 5 |
| Hypertension | 1 (2.3) | 1 | 1 (2.1) | 1 | 2 (4.9) | 2 | 1 (2.5) | 1 |  |  | 5 (2.3) | 5 |

ALT, alanine aminotransferase
